# A Tic-ing Time Bomb: Case Report of a Unique Presentation of Sudden-onset Tics

**DOI:** 10.5811/cpcem.20915

**Published:** 2025-01-16

**Authors:** Arino Neto, Vanessa Perez, Kim Manwaring, Lauren Averill, Victoria Wurster Ovalle

**Affiliations:** * Ann & Robert H. Lurie Children’s Hospital of Chicago, Division of Emergency Medicine, Chicago, Illinois; † Nemours Children’s Hospital, Florida, Division of Emergency Medicine, Orlando, Florida; ‡ Nemours Children’s Hospital, Florida, Division of Neurosurgery, Orlando, Florida; § Nemours Children’s Hospital, Delaware, Division of Radiology, Wilmington, Delaware

**Keywords:** Tics, arteriovenous malformation, parenchymal hemorrhage, hemorrhagic stroke

## Abstract

**Introduction:**

Tics in children are commonly diagnosed and not usually a cause for concern. Rarely, they may present as a symptom of underlying intracranial pathology.

**Case Report:**

We describe an adolescent with sudden-onset tics following a fall who presented to the emergency department and was diagnosed with an arteriovenous malformation with parenchymal hemorrhage. He underwent a successful embolization, after which his tics resolved.

**Conclusion:**

When evaluating a patient with tics, an atypical history or abnormal physical exam findings should raise suspicion for possible secondary etiologies, including arteriovenous malformation and stroke.

## INTRODUCTION

Tics are hyperkinetic movement disorders. They are repetitive, brief, and usually suppressible.^1^ Tics in children are well-recognized and typically not a cause for concern. When tic disorders arise abruptly or are persistent, secondary causes should be considered. Albeit infrequent, the development of a tic disorder following cranioencephalic trauma has been reported in the literature.^2^ We present a rare case demonstrating the importance of maintaining a broad differential diagnosis when a patient presents with sudden-onset tics.

## CASE REPORT

A 16-year-old male with autism and attention-deficit/hyperactivity disorder was referred to the emergency department (ED) for a new onset of tics following a head injury. Before the injury, the patient complained of malaise and diarrhea for several days. Approximately 24 hours before presenting to the ED, he was showering, briefly lost consciousness and fell, hitting the back of his head on the floor. He immediately had one episode of non-bilious, non-bloody emesis. Afterward, he returned quickly to his neurologic baseline. There was no external bleeding, bruising, abnormal movement, weakness, altered mental status, or fever.

**Image 1:**
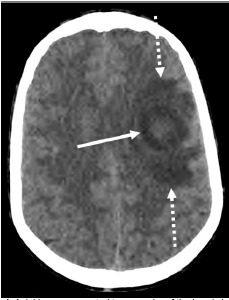
Axial image computed tomography of the head shows a rounded abnormality with mixed attenuation (solid arrow) in the left frontal lobe with surrounding hypodense vasogenic edema (dashed arrows).

The next day, the patient developed grimacing-type facial movements lasting one to two seconds at a time, occurring several times per hour. The patient was seen by his pediatrician and referred to the ED. Cardiovascular, pulmonary, and abdominal exams were unremarkable. On neurological evaluation, he was alert, oriented, and talking normally. The patient had no weakness or tremors. Cranial nerves II-XII were intact, and his gait was normal. However, he had frequent episodes of facial grimacing, occurring approximately every five minutes and lasting for about one second. Computed tomography (CT) of the head was obtained and showed a heterogeneous area of mixed attenuation in the left frontal lobe in the middle cerebral artery distribution (Image 1).

**Image 2:**
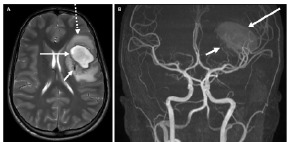
Magnetic resonance image (MRI) and angiogram of the head. (A) Axial T2-weighted MRI of the brain shows intraparenchymal hemorrhage in the left frontal lobe (long, solid arrow) with surrounding vasogenic edema (dashed arrow). Numerous tangled, low-signal vessels are seen posterior and medial to the hemorrhage, including a large draining vein (short arrow). (B) Coronal maximum intensity projection from time-of-flight magnetic resonance angiogram also shows the ovoid, hyperintense intraparenchymal hemorrhage (long arrow) as well as numerous abnormal feeding arteries (short arrow) indicative of an arteriovenous malformation arising from branches of the middle cerebral artery.

Neurology and neurosurgery services were consulted. The initial differential diagnosis included hemorrhagic stroke with underlying arteriovenous malformation (AVM) or tumor. An electroencephalogram was not performed. Nonetheless, the patient received levetiracetam for seizure prophylaxis given the concern for an intracranial process. Magnetic resonance imaging and angiography (MRI/MRA) studies of the brain were obtained, and he was admitted to the pediatric intensive care unit (PICU) for monitoring. The MRI/MRA of the brain showed a left posterior frontal lobe arteriovenous malformation with parenchymal hemorrhage and surrounding vasogenic edema (Image 2).

In the PICU, the patient received neuroprotective measures and remained stable. On day three of hospitalization, he was transferred to another hospital for an angiogram and endovascular neurosurgery assessment. He underwent a successful embolization of the AVM and started an intensive rehabilitation program. At three-month follow-up post procedure, the patient was doing well overall but had residual right-sided facial weakness and word-finding difficulties. He remains tic-free after the procedure.

**CPC-EM Capsule**

What do we already know about this clinical entity?

*Tics in children are common and typically do not warrant further workup. Arteriovenous malformations (AVM) are a rare cause of tics*.

What makes this presentation of disease reportable?

*We report a 16-year-old male who developed sudden-onset tics and was found to have an AVM. Only one other similar case appears in the literature*.

What is the major learning point?

*Patients with tics who present to the emergency department with an atypical history or abnormal physical exam findings may have an underlying cause (ie, acute intracranial pathology)*.

How might this improve emergency medicine practice?

*Emergency physicians have a crucial role in identifying concerning features in patients with neurological complaints that may warrant further workup*.

## DISCUSSION

Tic disorders may manifest in up to 10% of healthy children. They are commonly diagnosed between 3–10 years of age, are suppressible, and often there is a family history. The provisional tic disorder, formerly transient tic disorder, usually lasts for less than one year.^3^ Our patient was a 16-year-old male with no prior history of tics. Despite his overall well appearance and reassuring examination, the sudden onset of tics following his fall and loss of consciousness was concerning. His MRI/MRA showed a large left frontal AVM with initial concern for hemorrhagic stroke.

Reports in the literature of tic disorders developing after head trauma are rare.^2^ In a case series with 22 adult and three pediatric patients with emerging tics following traumatic brain injury (TBI), Ricketts et al found that 40% of the patients were diagnosed with damage, including hemorrhaging, to the basal ganglia, ventricular system, and temporal region. The authors concluded that tics emerging after TBI are relatively uncommon in youth and that it is challenging to confirm whether abnormal head imaging findings were pre-existing conditions.^4^ De Souza reported 13 cases of tics that developed after TBI. All but one patient was an adult, and four patients had positive, nonspecific findings on CT head or MRI brain. No cases of AVM or stroke were reported in this series.^5^

There are even fewer reports of AVM causing tics as the primary presenting symptom. Yochelson and David reported a 16-year-old male who presented with a complex tic disorder associated with vocalizations following a hemorrhage from a left frontal AVM.^6^ The patient was initially treated with carbamazepine due to concern for complex partial seizure. However, there were no changes to the movement disorder despite the therapeutic blood levels of antiseizure medication. His medication was transitioned to clonidine, and the frequency and severity of the tics had improved remarkably at six-month follow-up.

Neuroanatomical models propose that the pathophysiology of tics is related to basal ganglia and subthalamic dysfunction.^7^ There is a report in the literature of two eight-year-old boys presenting with Tourette syndrome-like symptoms and hemidystonia following right subcortical strokes involving the basal ganglia.^8^ A similar finding of vocal tics developing in a 71-year-old man following an acute lacunar infarct is also reported.^9^

We theorize that our patient had a non-traumatic rupture of his AVM, which led to a new-onset tic disorder. After evaluation by his pediatrician, the patient was referred to our ED, where we obtained a CT head. Our patient did not have classic signs of stroke, basal ganglia, or subthalamic dysfunction on neuroimaging. Nevertheless, we hypothesize that the hemorrhage from his AVM caused corticostriatal tract dysfunction, ultimately leading to basal ganglia activation and a hyperkinetic state.^7^,^8^ The patient’s tics improved after the embolization of his AVM, which also corroborates this neuroanatomical hypothesis for secondary tic development.

## CONCLUSION

We present a rare case of a hemorrhagic stroke due to arteriovenous malformation masquerading as a benign entity in a pediatric patient. Most tic disorders in the pediatric population are benign, transient, and require no treatment. However, when evaluating a patient with tics, certain aspects of the history and physical examination should raise concern for mimickers or underlying pathology, including AVM and stroke.
